# When the Tibia Hurts: Pain and Revision After Extendable Distal Femoral Endoprostheses in Young Patients

**DOI:** 10.1007/s43465-026-01768-4

**Published:** 2026-04-01

**Authors:** Muhammad Khatib, Assil Mahamid, Hamza Murad, Feras Qawasmi, Ali Yassin, Mustafa Yassin

**Affiliations:** 1 Department of Orthopedics, Hasharon Hospital, Rabin Medical Center, Affiliated to Tel Aviv University, 6997801 Tel Aviv, Israel; 2 Gray Faculty of Medical and Health Sciences, Tel Aviv University, Tel Aviv, Israel; 3 The Royal Orthopaedic Hospital NHS Foundation Trust, Bristol Road South, Northfield, Birmingham, UK; 4 Department of Orthopedics, Hillel Yaffe Medical Center, Hadera, Israel

**Keywords:** Bone remodeling, Sliding tibial component, Extendable distal femur endoprosthesis, Mid-shin pain

## Abstract

**Objective:**

En-bloc excision with limb salvage is the gold standard for aggressive bone tumors around the knee, but up to 15% of cases occur in skeletally immature patients, creating a risk of limb-length discrepancy. Growing distal femoral prostheses with passive sliding tibial components address this issue but introduce unique mechanical challenges and potential complications. This study aimed to evaluate the incidence of tibial pain and complications beneath extendable distal femoral endoprostheses, and to correlate clinical symptoms and revision surgery.

**Methods:**

The study comprised a retrospective review of 31 extendible distal femur endoprostheses from a single tertiary institution between 2008 and 2018. Measurements of radiographic parameters included coronal alignment, cortical thickness, cortical stem distances, stress shielding, and pedestal and periosteal reaction. The radiographic features were correlated with clinical evidence of tibial pain and the need for subsequent revision of the tibial component.

**Results:**

17 patients reported tibial pain during the follow-up period, with a mean time of onset of 62.2 months (range, 27–132). There were 14 revisions in 12 patients, 4 revisions for tibial pain. Stress shielding and pedestal formation were seen in all patients after 28 months following insertion. Lateral cortical hypertrophy was more prominent in the group with pain with a mean thickness of 5.8 mm (range, 4–9.8). Varus shift of the tibial stem was radiographically evident during follow-up (*n* = 18). 95% of the patients with tibial pain had radiographic evidence of stem migration, 88% showed a periosteal reaction, and 76% had varus malalignment. In 13 patients (76%) with pain, all three of these parameters were present.

**Conclusion:**

There is a strong correlation between radiographic evidence of tibial stem migration and periosteal reaction and the development of symptoms. Patients should be warned of the need for revision of the tibial component for pain during the lifetime of the implant.

**Level of evidence:**

IV.

## Introduction

Although primary malignant tumors of bone are rare in the general population, they are more common in the pediatric and adolescent populations, accounting for up to 15% of all malignancies in this age group. The distal femur is the most common site affected by primary malignant bone tumors. The optimal treatment for such tumors is en bloc excision, often in combination with neoadjuvant and adjuvant therapies, including chemotherapy, and in some cases, radiotherapy. Resection of the distal femoral physis compromises segmental growth of the affected limb, leading to the risk of limb-length discrepancy. Reconstruction options following *en bloc* resection in children include extendable endoprostheses, allografts, autografts, and distraction osteogenesis [1–4]. In the case of extendible endoprostheses, the extension module compensates for growth inconsistencies in patients who would otherwise develop an incapacitating leg discrepancy if given a fixed-length implant [5–8]. Extendable endoprostheses enable early weight-bearing, correction of a limb-length discrepancy, predictable function, psychological benefit, and reportedly lower risk of early complications [9, 10]. However, endoprostheses used in limb salvage have demonstrated limited longevity, resulting in high rates of reoperation due to mechanical failure and infection [11, 12].

Distal femoral endoprostheses consist of femoral and tibial components articulating at the knee in a rotating or non-rotating hinge joint. The femoral stem is either cemented or uncemented, and most designs incorporate a collar coated with hydroxyapatite to improve osseointegration and reduce the incidence of aseptic loosening [13]. On the tibial side, constructs often utilize a passive sliding component that is not integrated into native bone, allowing for continued growth from the proximal tibial physis. To enable growth of the proximal tibia, the tibial component of some implants consists of a polyethylene sleeve fixed onto the proximal tibia with a metallic stem sliding through the sleeve [14].

Earlier studies have compared bone remodeling around cemented and uncemented stems in growing prostheses [14, 15]. *Blunn *et al*.* [15] described seven cases with histological and radiographical remodeling around cemented femoral stems and cemented sleeves with sliding tibial stems. *Jaiswal *et al. [14] demonstrated no correlation between clinical, pathological, and radiological outcomes when comparing survival rates and radiographic parameters between groups with and without polyethylene sleeves.

Furthermore, patients report experiencing shin pain after undergoing revision total knee arthroplasty with stemmed components [16, 17]. Similarly, end-of-stem pain and thigh pain have been described for cementless total hip replacements [18–22]. Recently, studies about extendable distal femur prostheses with passive sliding tibial stems have not mentioned the issue of tibial pain and resulting revision rates. The Henderson classification is the most commonly used method for classifying the modes of failure of reconstruction techniques, including endoprosthetic replacement, following resection of bone for malignant bone tumors (REF). In 2014, this system was modified to include modes of failure following extendible prostheses [23]. In this modified system, physeal arrest and joint dysplasia were classified as type 6 failures. However, revision due to pain arising from the passive tibial component remains unclassified.

Accordingly, this study aimed to comprehensively evaluate the clinical outcomes and revision rates of extendable distal femoral endoprostheses. Particular emphasis was placed on characterizing radiographic changes in the tibia following the insertion of a sliding tibial stem, assessing the incidence of mid-shin pain, and determining its potential association with implant survival and the need for revision surgery.

## Materials and Methods

### Study Design and Setting

This retrospective study was conducted at a single tertiary referral orthopedic oncology unit and included all patients treated for a histologically confirmed primary malignant bone tumor of the distal femur between 2008 and 2018. During the study period, all implanted devices were of comparable design, and all patients were followed for a minimum of 18 months after surgery. Patients were identified through a prospectively maintained institutional database that captured all relevant diagnostic, treatment, and follow-up data.

### Patient Identification and Radiographic Assessment

Only patients who were managed entirely through the orthopedic oncology unit from the time of diagnosis were eligible. Those who were diagnosed or treated elsewhere and subsequently referred for a second opinion were excluded. Additional exclusion criteria were the absence of a passive sliding tibial stem, radiological or clinical follow-up of less than 18 months, inadequate radiographs precluding measurement, or implantation performed as revision surgery. Complete clinical, tumor, and radiographic records were available for all included patients, ensuring a comprehensive dataset for analysis. Radiographic assessments were conducted by senior orthopedic surgeons with specialized expertise in knee megaprosthesis surgery; however, the evaluations were not performed in a blinded manner. Stem migration was assessed using serial radiographic analysis on standardized anteroposterior and lateral knee radiographs. A continuous longitudinal line was drawn along the central axis of the tibial stem, and its distal intersection with the adjacent bone was identified on both projections to assess angular deviation over time. In addition, linear distances from the tip of the tibial stem to the medial and lateral tibial cortices were measured on AP radiographs to detect asymmetric migration or varus–valgus drift. Measurements were obtained at two time points: the first postoperative follow-up and the final follow-up, and changes between these assessments were interpreted as evidence of stem migration. No absolute cut-off value was predefined; instead, migration was evaluated based on temporal progression and clinical relevance.

### Indications for Reconstruction

A growing distal femoral endoprosthesis was indicated for skeletally immature patients under the age of 13 years with a predicted additional growth of at least 2 cm prior to physeal closure or skeletal maturity. Each case was reviewed by a multidisciplinary sarcoma team, and the method of reconstruction was tailored to the individual patient.

### Surgical Technique

Reconstruction in all cases consisted of a customized growing distal femoral endoprosthesis (Stanmore Implants Worldwide, Elstree, London, UK). The growing mechanism was either noninvasive, using an extracorporeal magnetic distraction device, or minimally invasive, using a cam and screw system accessible through a small arthrotomy. All femoral components were cemented in place with Palacos R bone cement (Heraeus Medical GmbH, Germany). Following tumor resection, the proximal tibial surface was carefully resected to avoid damage to the underlying physis, and a central medullary perforation was created to accommodate the sliding tibial component. The proximal tibia was prepared to permit impaction of the polyethylene sleeve, which was inserted within the medullary canal and secured with a thin layer of bone cement to provide rotational stability. The passive sliding tibial component was then inserted through the sleeve, engaging with the femoral condyles to form a rotating hinge prosthesis secured with an axle. In cases where the proximal tibia was too small to accept a sleeve, the sliding tibial component was inserted and secured without one.

### Statistical Analysis

All data management and statistical analyses were performed using Python (version 3.11) with the pandas, NumPy, SciPy, and matplotlib libraries. Continuous variables were expressed as means with standard deviations (SD) or medians with interquartile ranges (IQR), as appropriate. Categorical variables were summarized as frequencies and percentages. Comparisons between patients with and without tibial pain were conducted using the Fisher’s exact test or Chi-square test for categorical variables, and the independent *t* test for continuous variables. A *p* < 0.05 was considered statistically significant.

Radiographic alignment was analyzed at two timepoints immediately postoperative and final follow-up to evaluate longitudinal changes. Proportional differences between neutral and varus alignment were illustrated using a bar chart. Time-dependent occurrence of tibial pain was assessed using Kaplan–Meier survival analysis, with months from implantation to pain onset as the event variable. Median time to tibial pain onset and interquartile range were reported.

### Implant Survival and Growth Analysis

The incidence and indication for revision were recorded to determine implant survival. Tibial length between the proximal and distal physes on the most recent radiograph was measured to evaluate tibial growth and to assess the ability of the passive sliding stem to maintain limb length.

### Ethical Approval

The study protocol was reviewed and approved by the Institutional Review Board of the center. Owing to the retrospective design and reliance on existing electronic records, the requirement for informed consent was waived. AI-based tools were used exclusively to improve grammar, clarity, and English language style during manuscript preparation. These tools were not employed for data analysis, statistical evaluation, or interpretation of results.

## Results

A total of 31 pediatric patients were included in the analysis, comprising 18 females (58.1%) and 13 males (41.9%), with a mean age of 10.8 ± 2.1 years (range, 7–15 years). In Table 1, the predominant diagnosis was high-grade osteoblastic osteosarcoma (87.1%), followed by chondroblastic osteosarcoma (6.5%), parosteal osteosarcoma (3.2%), and spindle cell/other histologies (3.2%). The majority of reconstructions were performed on the right side (77.4%), with the remaining 22.6% on the left.

**Table 1:** Demographic and clinical characteristics of pediatric patients undergoing expandable distal femoral endoprosthetic reconstruction

Variable	Category	*N*	%
Gender	Female	18	58.1
	Male	13	41.9
Age (years)	Mean ± SD (range)	10.8 ± 2.1 (7 – 15)	–
Diagnosis	High-grade osteoblastic osteosarcoma	27	87.1
	Chondroblastic osteosarcoma	2	6.5
	Parosteal osteosarcoma	1	3.2
	Spindle cell/other	1	3.2
Side	Right	24	77.4
	Left	7	22.6
Follow-up (months)	Mean ± SD (range)	90.1 ± 39.5 (18–172)	–
	Median (IQR)	87 (54–125)	–
Revision surgery	Yes	14	45.2
	No	17	54.8
Grower type	Non-invasive	23	74.2
	Ext/minimal-invasive	8	25.8
Pain reported	Mid-shin pain	17	54.8
	No pain	14	45.2
Mortality		5	16.1

The mean follow-up duration was 90.1 ± 39.5 months (median 87 months, interquartile range 54–125). Fourteen patients (45.2%) underwent at least one revision procedure, while 17 (54.8%) had no revision during the study period. Most implants were noninvasive growing prostheses (74.2%), whereas 25.8% were extensible or minimally invasive devices. Mid-shin pain was reported in 17 patients (54.8%), while 14 (45.2%) remained asymptomatic throughout follow-up. The overall mortality rate at final follow-up was 16.1% (*n* = 5). Beyond stem-related tibial pain, no consistent additional knee-level clinical symptoms, including instability, mechanical locking, or extensor mechanism dysfunction, were documented during follow-up.

In Table 2, radiographic evaluation demonstrated distinct differences between patients with and without tibial pain. Postoperatively, most implants were aligned neutrally in both groups (88% overall), with no significant difference in alignment immediately after surgery (*p* = 0.37). However, at the last follow-up, varus alignment was significantly more prevalent among patients who developed tibial pain (76%) compared with those without pain (36%) (*p* = 0.033). No clinically relevant multiplanar deformities, including sagittal plane abnormalities such as knee flexion deformity, were observed during follow-up; all documented deformities were confined to the coronal plane.

**Table 2:** Radiographic parameters and their association with tibial pain following expandable distal femoral endoprosthetic reconstruction

Radiographic parameter	Tibial pain (*n* = 17)	No tibial pain (*n* = 14)	All (*n* = 31)	*p* value
Component alignment (postoperative)—neutral	15	10	25	0.370
Component alignment (postoperative)—varus	2	4	6	0.370
Component alignment (last follow-up)—neutral	4	9	13	0.033
Component alignment (last follow-up)—varus	13	5	18	0.033
Stress shielding	17	10	27	0.032
Pedestal sign	17	8	25	0.004
Resorption of the epiphysis	5	2	7	0.412
Stem migration	16	7	22	0.011
Periosteal reaction	15	3	18	< 0.001

^*^A *p* value of < 0.05 was considered statistically significant

Radiographic evidence of altered load transfer around the tibial component was highly prevalent in the study cohort. Stress shielding was observed in 27 of 31 patients (87.1%) overall and occurred significantly more frequently in patients who reported tibial pain compared with those without pain (100% vs. 71.4%, *p* = 0.032). This finding suggests a strong association between proximal tibial bone demineralization and clinically relevant stem-related symptoms. Similarly, the pedestal sign, characterized by distal cortical hypertrophy or endosteal bone formation at the tip of the tibial stem, was identified in 25 patients (80.6%) and was significantly more common in the tibial pain group than in asymptomatic patients (100% vs. 57.1%, *p* = 0.004). The presence of a pedestal sign frequently coexisted with stress shielding and stem migration, indicating a non-physiological distal load concentration at the stem tip.

Stem migration occurred in 71% of painful cases versus 50% of asymptomatic cases (*p* = 0.011). Periosteal reaction was noted in 83% of patients with pain compared with only 21% of those without pain, showing a highly significant association (*p* < 0.001). Resorption of the epiphysis was uncommon and did not differ significantly between groups (*p* = 0.412).

**Fig. 1:**
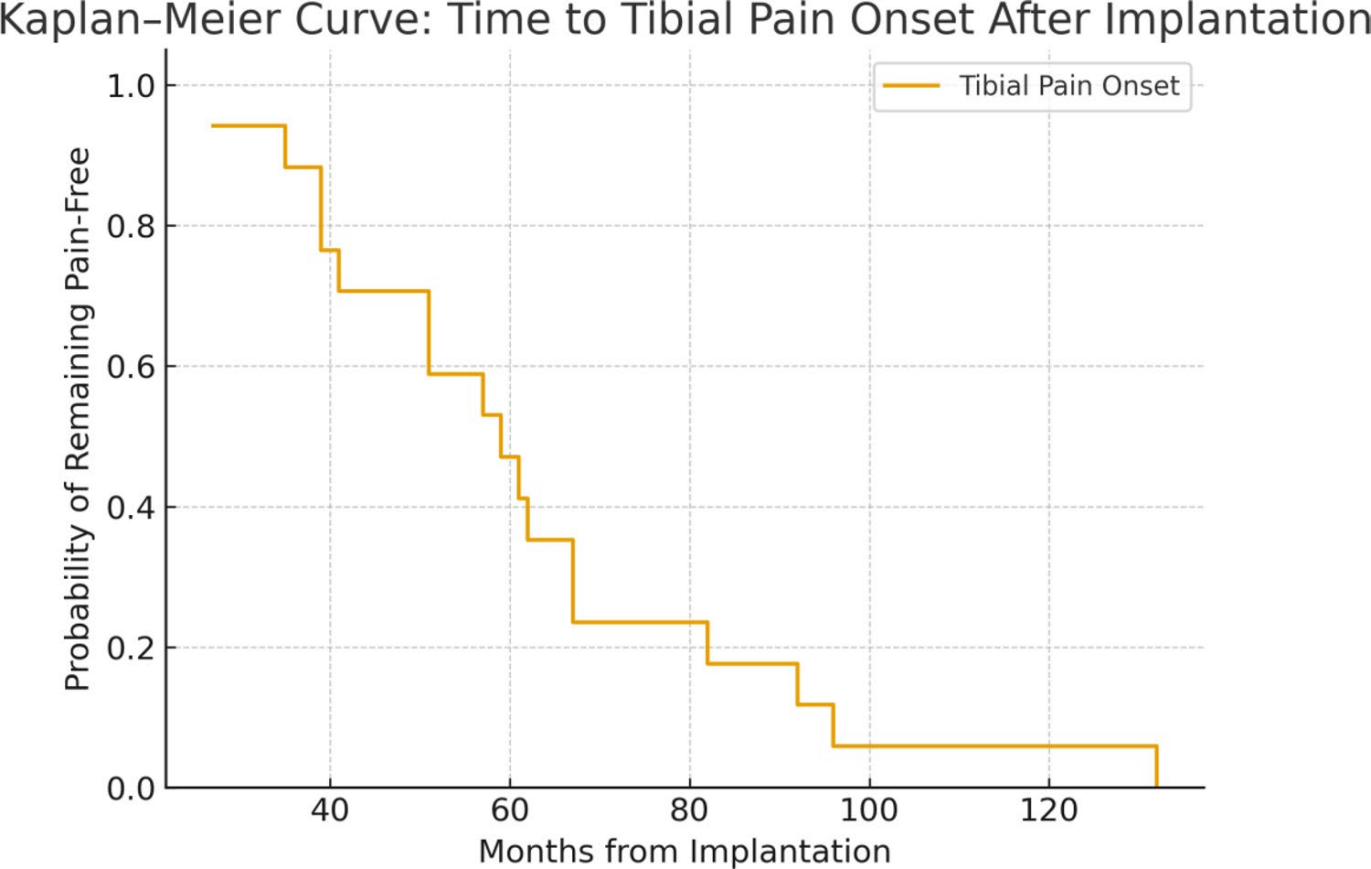
Kaplan–Meier curve demonstrating time to tibial pain onset following expandable distal femoral endoprosthetic reconstruction

In Fig. 1, Kaplan–Meier analysis demonstrated a progressive decline in pain-free survival over time following implantation. The median time to tibial pain onset was 62 months (interquartile range, 39–92 months). Approximately 25% of patients reported pain within the first 40 months post-implantation, and by 80 months, more than 75% had developed tibial discomfort. The probability of remaining pain-free decreased sharply between 50 and 90 months, indicating a delayed but cumulative onset pattern. No censoring occurred before 10 years of follow-up, suggesting consistent long-term surveillance across the cohort. No coronal plane deformity was present in the study cohort; therefore, no growth modulation of the distal tibial or fibular physis was performed, and surgical management was directed exclusively toward limb-length restoration.

Among the 31 patients, a total of 14 revision procedures were performed, including two patients who underwent multiple revisions. In Table 3, the most frequent causes were maximal lengthening (28.6%) and mid-shin pain (28.6%), followed by femoral stem fracture (14.3%) and failure of the growing mechanism (14.3%). Less common indications included prosthetic joint infection (7.1%), prosthetic fracture (7.1%), aseptic loosening (7.1%), and retropatellar pain (7.1%). Mechanical complications accounted for the majority of revision procedures, emphasizing the long-term structural vulnerability of expandable prosthetic constructs in pediatric patients.

**Table 3:** Causes of revision in patients with expandable distal femoral endoprostheses

Cause of revision	Number of cases (*n*)	% of total revisions (*n* = 14)
Maximal lengthening	4	28.6
Mid-shin pain	4	28.6
Femoral stem fracture	2	14.3
Failure of growing mechanism	2	14.3
Prosthetic joint infection (PJI)	1	7.1
Prosthetic fracture	1	7.1
Aseptic loosening	1	7.1
Retropatellar pain	1	7.1
Total revisions*	14	–

^*^Includes 2 patients who underwent two separate revision procedures

**Fig. 2:**
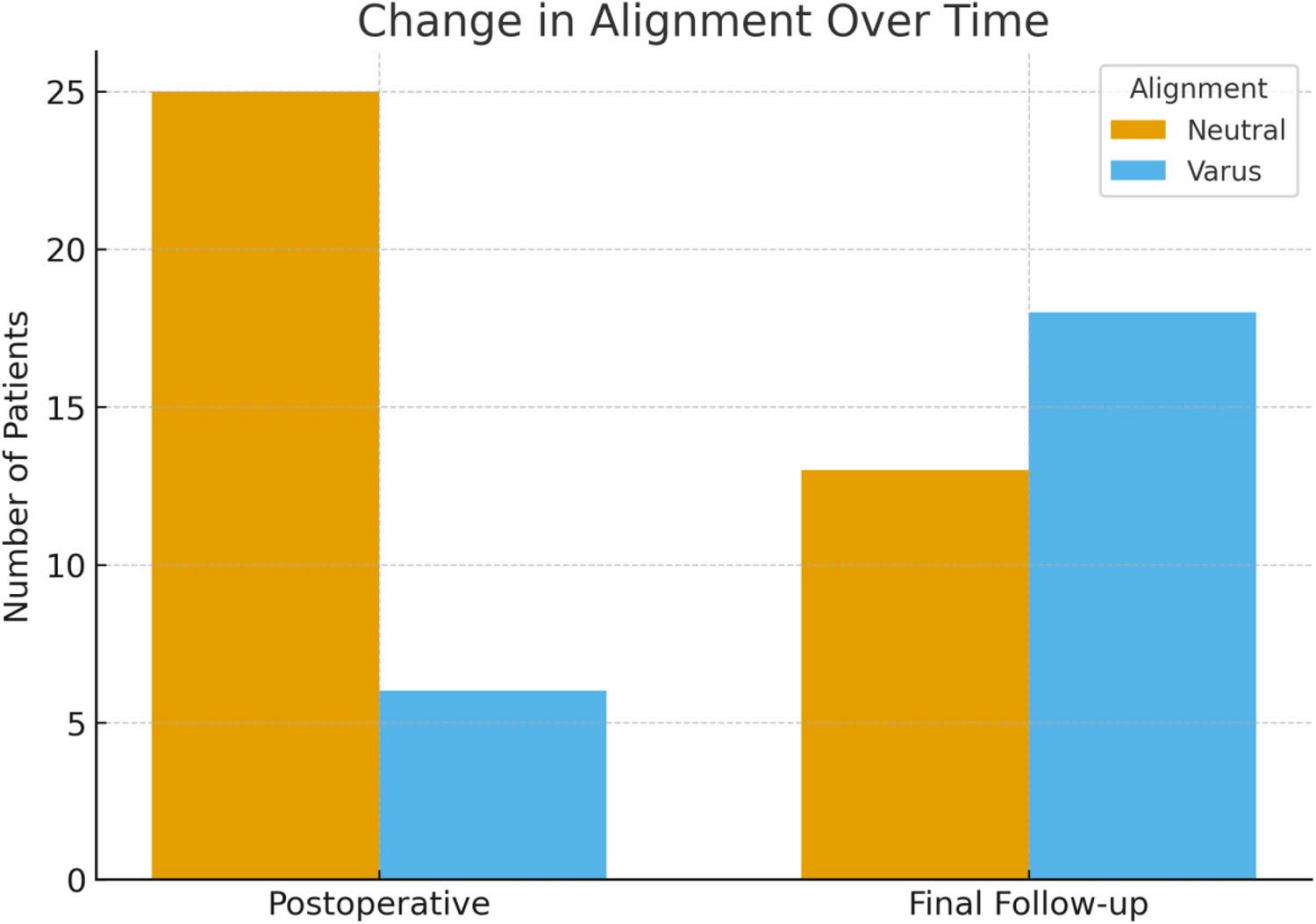
Change in component alignment between postoperative and final follow-up evaluations

In Fig. 2, radiographic analysis revealed a clear shift toward varus component alignment over the follow-up period. Immediately postoperatively, 25 implants (81%) were positioned neutrally and 6 (19%) in varus. At the final follow-up, neutral alignment decreased to 13 implants (42%), while varus alignment increased to 18 implants (58%). This progressive varus trend suggests a tendency for gradual mechanical deviation over time, likely reflecting bone remodeling and load redistribution around the growing prosthetic construct. At final follow-up, tibial length discrepancy was calculated as the absolute difference between preoperative tibial length and final postoperative tibial length after completion of the lengthening process. Among patients with available measurements (*n* = 23), the mean final tibial length discrepancy was 1.76 cm (range, 0.10–4.02 cm). Resurgery related to maximal lengthening was observed in four cases and was uniformly associated with excessive postoperative pain. In all affected patients, the achieved tibial lengthening exceeded 2 cm, which emerged as a consistent clinical threshold beyond which pain became significant and refractory to conservative measures, including analgesia and activity modification.

## Discussion

In this retrospective series of 31 pediatric patients (mean age 10.8 years) treated with extendable distal femoral endoprostheses, high-grade osteoblastic osteosarcoma represented the predominant indication. Over a mean follow-up of 90 months, nearly half of the cohort (45.2%) required at least one revision, while mid-shin pain developed in 54.8% of patients after a median of 62 months. Radiographic assessment demonstrated progressive varus deviation over time, with stress shielding (87%), pedestal formation (100% vs. 57%; *p* = 0.004), stem migration (71% vs. 50%; *p* = 0.011), and periosteal reaction (83% vs. 21%; *p* < 0.001) all significantly associated with tibial pain. Conversely, epiphyseal resorption was infrequent and not pain-related (*p* = 0.412). Mechanical complications primarily maximal lengthening and mid-shin pain were the leading causes of revision, underscoring the structural vulnerability and long-term biomechanical challenges of expandable prosthetic constructs in skeletally immature patients.

The true incidence of tibial pain after growing distal femoral endoprosthetic replacement is not well documented. Existing studies in skeletally immature patients primarily report tibial growth disturbance and multiplanar deformities, with rates as high as 70% in some series. Although these reports rarely quantify pain, it is frequently associated with deformity and leg-length discrepancy, suggesting a shared pathophysiological mechanism [24, 25].

Stress shielding and the pedestal sign were among the most prevalent radiographic findings observed in tibiae reconstructed with passive sliding stems. In the present cohort, stress shielding was identified in 27 patients (87%) and was significantly associated with tibial pain (*p* = 0.032). Similarly, the pedestal sign was markedly more frequent among patients with pain (100% vs. 57%; *p* = 0.004), suggesting that these radiological findings may reflect clinically relevant alterations in load transfer and stem–bone interaction.

Experimental, biomechanical, and computational studies consistently demonstrate that long, rigid metallic stems, particularly cobalt–chromium components, produce non-physiological stress distribution within the proximal tibia and distal femur, resulting in localized bone remodeling adjacent to the implant [26–29]. Despite this well-established biomechanical rationale, the clinical literature specifically addressing the incidence and implications of stress shielding in knee megaprosthesis populations remains limited. Available systematic reviews and retrospective series acknowledge stress shielding as a potential complication but do not report precise incidence rates; it is generally considered less frequent than infection or aseptic loosening, yet clinically relevant [30, 31].

Similarly, the pedestal sign has not been systematically quantified in knee megaprosthesis cohorts and is not emphasized as a major complication in large series or systematic reviews, which predominantly focus on infection, mechanical failure, and aseptic loosening as the leading causes of revision [30–32]. This discrepancy between reported literature and the high prevalence observed in the present cohort suggests that such radiographic changes may be underrecognized or underreported in long-term follow-up studies.

Although both stress shielding and pedestal sign were significantly associated with tibial pain in the current analysis, prior studies have questioned their direct clinical relevance, as these findings often emerge relatively early in the postoperative course, whereas tibial pain tends to develop later. Consistent with the existing literature, many patients with these radiographic findings remain asymptomatic, indicating that their presence alone is insufficient to predict pain or implant failure [33–35]. Notably, the significantly higher rates of stem migration and periosteal reaction observed among symptomatic patients suggest that progressive biomechanical adaptation or micro-instability may represent a critical factor in the transition from radiographic changes to clinical symptoms.

The femoral component of extendible distal femoral megaprostheses appears to be more susceptible to structural failure than the tibial component, largely due to biomechanical and implant design-related factors. The femoral stem is exposed to greater mechanical loads, particularly in long segmental reconstructions, where increased lever arms and bending moments amplify stress across the implant–bone interface. This vulnerability is further accentuated when smaller intramedullary stem diameters are combined with longer extramedullary components, a configuration that has been shown to significantly increase the risk of femoral stem fracture. [36] In addition, the presence of screw holes within the femoral stem creates stress concentrations that may serve as initiation sites for fatigue crack propagation, thereby further predisposing the femoral component to mechanical failure.

In contrast, the tibial component, whether metal-backed or all-polyethylene, rarely fails due to fracture. Clinical series demonstrate high survival rates for tibial components and low rates of mechanical failure, with most complications related to infection or loosening rather than structural fracture. [2] The tibial component is generally less exposed to the same magnitude of bending and torsional forces as the femoral stem, and its design does not typically include features such as screw holes that act as stress risers.

Previous studies have described the impact of the polyethylene sleeve on tibial outcomes. One study described that physeal growth continues despite the presence of a defect caused by the polyethylene sleeve and metal stem [23]. Furthermore, another study reported that the presence of this polyethylene sleeve is associated with lateral migration of the tip of the tibial prosthesis, and therefore, cortical reaction seen at the tip of the tibial prosthesis is a consequence of its migration [14]. Finally, one more study showed that a region of sclerotic bone usually develops around the tapped polyethylene sleeves, and that sclerotic bone has also developed under the tibial plateau [15].

In our study, 71% of the patients with epiphyseal resorption (7) suffered from pain, all of whom showed evidence of stem migration and varus malalignment at their last follow-up. The resorption may indicate vascular damage of the epiphysis during the tibial resection, either due to the cement used to bond the polyethylene sleeve, or damage caused by canal preparation. This resorption may be a contributing factor to subsequent stem migration and varus malalignment.

Resurgery attributable to maximal lengthening was required in four cases and was consistently associated with significant postoperative pain. In all affected patients, the achieved tibial lengthening exceeded 2 cm, which emerged as a reproducible clinical threshold beyond which pain became clinically meaningful and refractory to conservative management, including analgesic therapy and activity modification. Consequently, the indication for reintervention was determined by the combination of exceeding this lengthening threshold and the presence of persistent, function-limiting pain, rather than radiographic findings alone. These findings suggest that tibial lengthening beyond 2 cm may surpass the adaptive capacity of the surrounding soft tissues in this reconstructive context, underscoring the importance of meticulous intraoperative planning and vigilant postoperative surveillance.

**Fig. 3:**
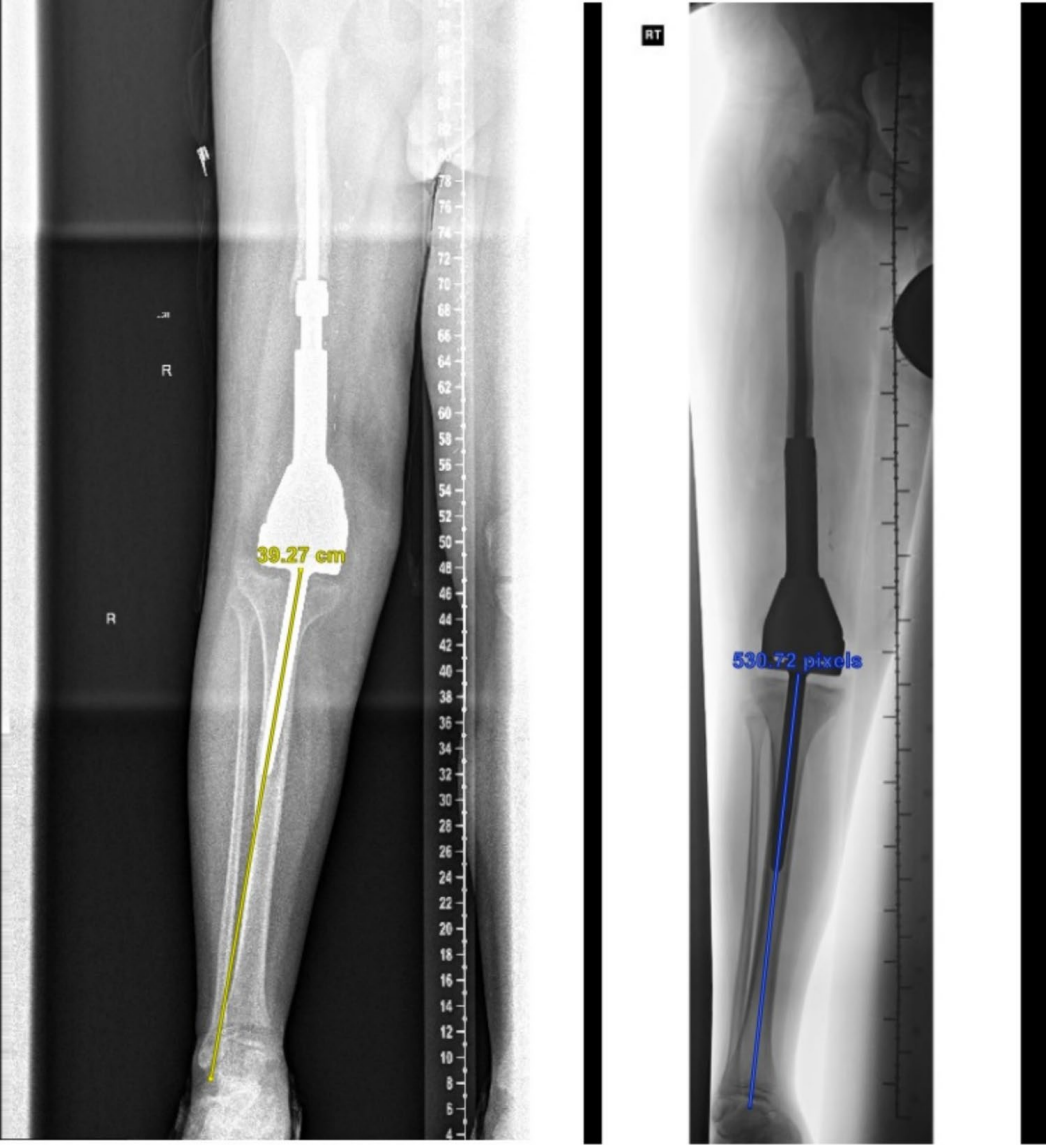
A 9 year-old patient followed from 2013 to 2019, demonstrating progressive varus deformity with lateral migration of the tibial stem tip, associated with mid-shin pain

**Fig. 4:**
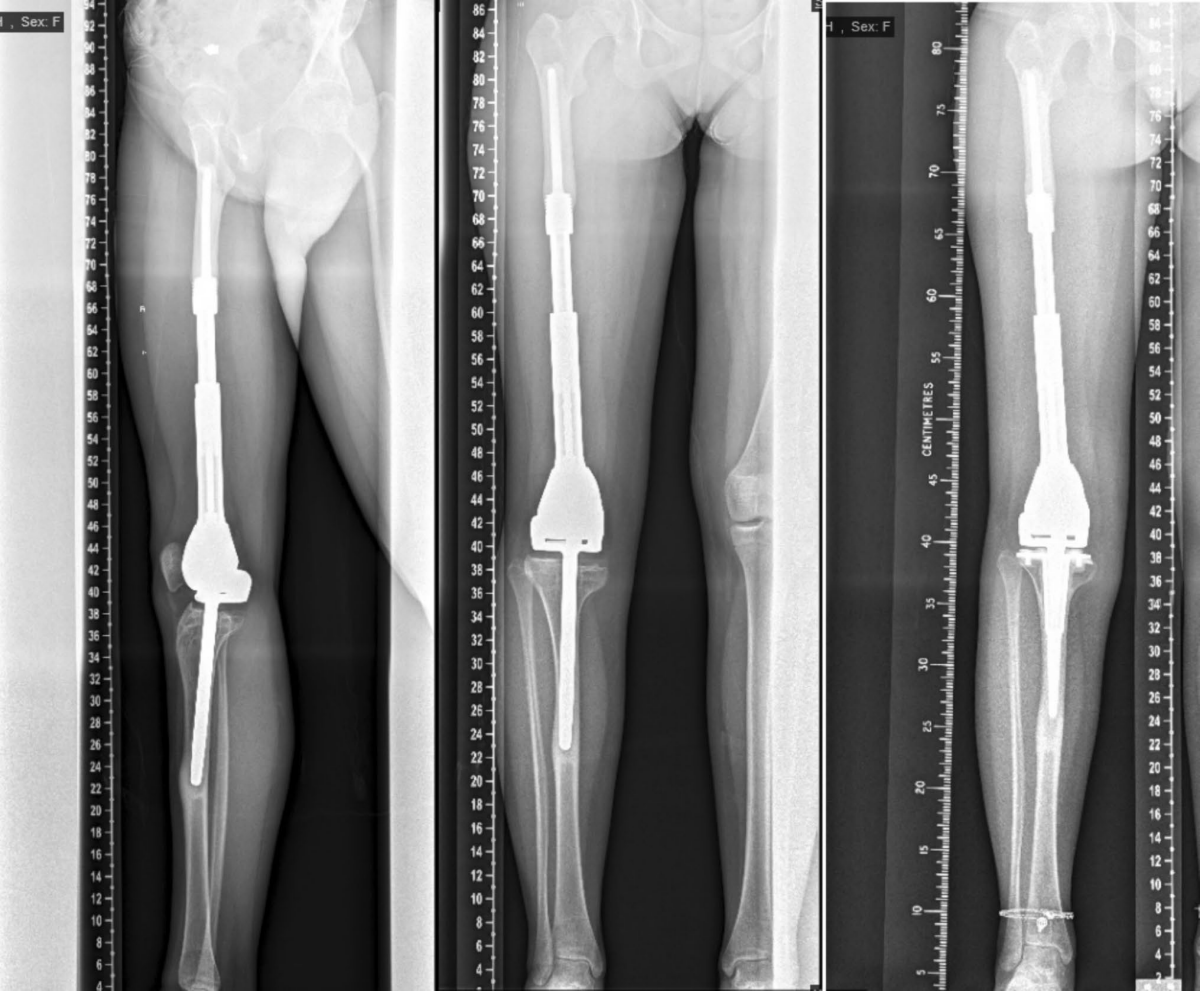
A 14 year-old female, five years postoperatively, demonstrating cortical hypertrophy, periosteal reaction, pedestal formation, resorption of the medial epiphysis, and evidence of stress shielding. The patient underwent revision surgery in 2019 with cemented tibial fixation due to persistent tibial pain

**Fig. 5:**
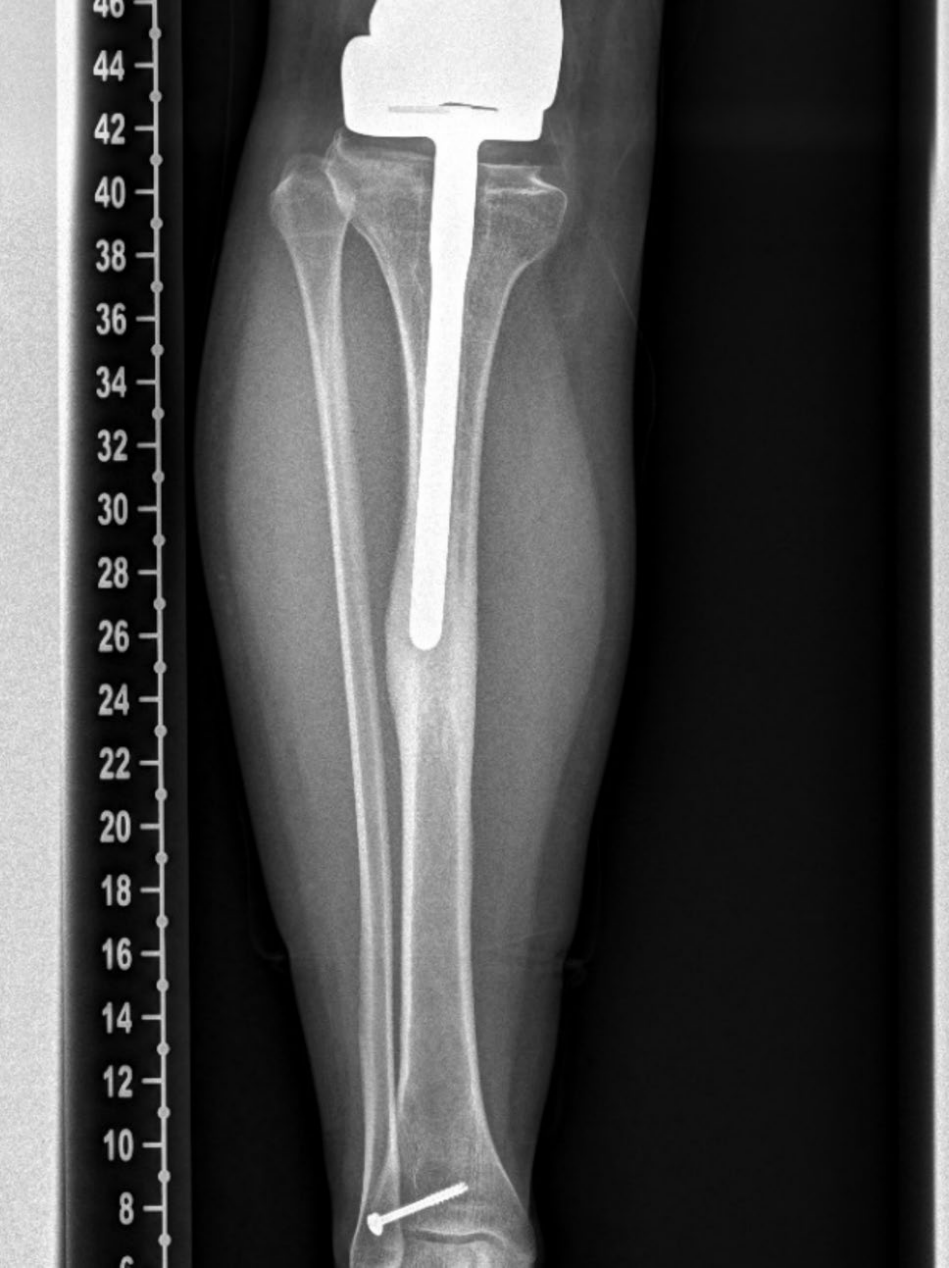
An 18 year-old female, eight years following expandable distal femoral endoprosthesis reconstruction, demonstrating marked cortical hypertrophy, progressive stem migration with varus deformity, and periosteal reaction on plain radiographs

Stem migration was strongly associated with progressive varus deformity and changes in tip-to-cortex distance, with varus malalignment appearing to be the main contributor, as most stems were already near the lateral cortex on initial radiographs (Fig. 3). Migration, periosteal reaction, and varus alignment were the radiographic parameters most closely linked to tibial pain, present in 94%, 88%, and 76% of symptomatic patients, respectively, with most demonstrating all three concurrently (Fig. 4). The cortical hypertrophy and periosteal reaction likely represent adaptive responses to mechanical instability, modulus mismatch, and altered loading caused by stem migration (Fig. 5).

A key strength of this study lies in its inclusion of the largest known cohort of pediatric patients treated with extendable distal femoral endoprostheses to date. The extensive follow-up period, coupled with comprehensive radiographic and clinical evaluation, provides robust insight into the long-term mechanical behavior and survivorship of these complex constructs. By correlating radiographic remodeling patterns, such as stress shielding, pedestal formation, and stem migration with clinical outcomes, including mid-shin pain and revision rates, this study offers a uniquely detailed understanding of implant–bone interaction and failure mechanisms in growing endoprosthetic systems.

Varus collapse remains a recognized mechanical failure mode following extendable distal femoral endoprosthetic reconstruction, particularly in the setting of extensive bone loss and high mechanical demands [37]. Emerging evidence suggests that both implant design and fixation methodology play a pivotal role in mitigating this complication. Stem geometry should be tailored to the defect location and femoral canal morphology, with tapered or conical stems offering improved circumferential contact, load distribution, and stability in larger or more proximal defects, while alternative geometries may be suitable for more distal reconstructions [38, 39]. Equally important is the achievement of reliable metaphyseal fixation, as metaphyseal sleeves or cones enhance rotational stability and load transfer and have been shown to reduce early aseptic loosening, particularly when distal femoral augmentation exceeds critical thresholds [40]. Zonal fixation strategies that integrate diaphyseal stem anchorage with metaphyseal support are therefore recommended to minimize micromotion and asymmetric varus loading [37, 40, 41]. Although current recommendations are largely derived from biomechanical studies and retrospective clinical series, they underscore the importance of meticulous preoperative planning, implant selection, and fixation strategy to reduce the risk of varus failure in this challenging reconstructive context.

Our study has several limitations that merit consideration. As a retrospective analysis, it is inherently limited by the data available and cannot fully establish causality between the radiographic findings and the development of symptoms. Although we used a prospectively maintained database, certain details, such as pain severity and functional outcome scores, were not consistently documented. The study was performed at a single tertiary referral center, which may limit how broadly these results can be applied to other settings or implant designs. The relatively small sample size reflects the rarity of this patient population but may have limited our ability to detect more subtle associations. Radiographic evaluation was based on standardized plain films, which may not completely capture the three-dimensional nature of tibial alignment or cortical remodeling. The radiographs included were illustrative rather than representative of the full cohort, and long-term images demonstrating preserved epiphyseal growth were not available. Additionally, the retrospective design limited comprehensive assessment of radiographic evolution over time. Moreover, the lack of blinded radiographic assessment represents a limitation of this study; however, evaluations were performed by experienced surgeons specializing in knee megaprosthesis reconstruction, which likely mitigated interobserver variability and enhanced the reliability of the assessments. Finally, while our minimum follow-up of 18 months was adequate to capture most clinically relevant events, longer-term data will be needed to understand the full natural history of these complications over time.

## Conclusion

Extendable distal femoral endoprostheses with passive sliding tibial stems remain an essential solution for limb salvage in skeletally immature patients; however, they are associated with a considerable incidence of tibial pain and revision surgery. Our findings suggest that pain is not primarily related to early radiographic changes such as stress shielding or pedestal formation but rather follows a gradual sequence beginning with physeal damage, progressing through cortical hypertrophy and varus malalignment, and culminating in stem migration and periosteal reaction. These results highlight the need for closer surveillance of alignment and stem position during follow-up and suggest that pain-related failure should be explicitly incorporated into future iterations of the Henderson classification to better capture the clinical significance of this complication.
